# Mechanisms of Anticancer Activity of a Fatty Acid Mixture Extracted from Hen Egg Yolks Enriched in Conjugated Linoleic Acid Diene (CLA) against WM793 Melanoma Cells

**DOI:** 10.3390/nu13072348

**Published:** 2021-07-09

**Authors:** Dominik Domagała, Teresa Leszczyńska, Aneta Koronowicz, Barbara Domagała, Mariola Drozdowska, Ewelina Piasna-Słupecka

**Affiliations:** 1 Department of Human Nutrition and Dietetics, Faculty of Food Technology, University of Agriculture in Krakow, 30-149 Krakow, Poland; teresa.leszczynska@urk.edu.pl (T.L.); aneta.koronowicz@urk.edu.pl (A.K.); mariola.drozdowska@urk.edu.pl (M.D.); ewelina.piasna@urk.edu.pl (E.P.-S.); 2 Department of Horticulture, Faculty of Biotechnology and Horticulture, University of Agriculture in Krakow, 31-425 Krakow, Poland; barbara.domagala@urk.edu.pl

**Keywords:** apoptosis, cancer, CLA, conjugated linoleic acid dienes, functional foods, melanoma

## Abstract

The conjugated linoleic acid (CLA) diene is a biologically active compound with proven health-promoting effects. In terms of anticancer properties, it has been shown that CLA reduces the proliferation of cancer cells. In this study, it has been demonstrated that a mixture of fatty acids, isolated from chicken egg yolk enriched in CLA isomers by biofortification, reduces (by 30.5%) the proliferation of human melanoma cancer cells line WM793 to a greater extent than a mixture of fatty acids not containing these isomers. At the same time, the tested fatty acid mixtures show no effect on human normal BJ fibroblast cells. For the first time, the genes with increased expression have been identified and the proteins have been activated by the fatty acid mixture of CLA-enriched egg yolk, mainly responsible for mitochondrial pathway-dependent apoptosis.

## 1. Introduction

Cancer is a group of diseases characterised by uncontrolled cell growth and the ability to infiltrate other parts of the body. It arises from the transformation of normal cells to cancer cells in a multistep process [1]. It is believed that 10–15% of all cancers are related to heredity and the remaining 85–90% of cases are rooted in the environment and lifestyle. The most important environmental factors that increase cancer risk include smoking (25–30%), diet (30–35%), infections (15–20%), radiation (up to 10%), as well as stress, physical inactivity, and environmental pollution [2].

Changes in dietary habits, including the consumption of functional foods with proven anti-cancer properties, may be an important factor in reducing the risk of the onset and development of these diseases.

Conjugated linoleic acid (CLA) diene is a mixture of positional and geometric isomers of linoleic acid (C18:2, LA—linoleic acid), with two conjugated unsaturated double bonds occurring at different positions of the fatty acid carbon chain [3]. Of all the CLA isomers, two of them are of greatest physiological importance: cis-9, trans-11 CLA and trans-10, cis-12 CLA [4]. CLA has anti-diabetic, anti-atherosclerotic and anti-cancer properties [5,6,7].

The available literature lacks data on the mechanisms of apoptosis of melanoma cancer cells of line WM793 induced by a mixture of egg yolk fatty acids biofortified with CLA.

The aim of the present study is to determine for the first time, in vitro, the unstudied mechanisms of the potentially anticancer effects of a mixture of fatty acids, extracted from hen egg yolks enriched in conjugated linoleic acid (CLA) diene, on human cancerous melanoma cell line WM793.

## 2. Materials and Methods

### 2.1. Enrichment of Eggs in CLA and GCMS Analysis of Extracted Fatty Acids

The production process of chicken eggs biofortified in conjugated linoleic acid diene, the extraction of fatty acids, as well as their chromatographic separation and identification were described in the previous study by Koronowicz et al. [8]. Briefly, to obtain CLA-enriched eggs, Isa Brown laying hens were fed a modified “DJ” mix with 0.75% CLA for 4 months. The source of CLA isomers was the commercial preparation TonalinFFA 80 (80% CLA, BASF, Ludwigshafen, Germany), which contained, as active substances, two CLA isomers: cis-9, trans-11 and trans-10, cis-12 in the proportion 50:50. The control group were hens fed with standard “DJ” feed without CLA supplements. Next the egg yolks were frozen (−20 °C) and subjected to a freeze-drying process (freeze drying dryer: Martin Christ Model Alpha 1–4, Osterode am Harz, Germany).

Extractions of fatty acids from freeze-dried chicken yolks were performed by the Folch method [9] using a chloroform-methanol mixture (2:1) with the addition of 1 g/L of butylated hydroxytoluene (Sigma-Aldrich, St. Louis, MO, USA). The extracted fatty acids were dissolved in ethanol prior cell treatment. The final concentration of ethanol in the culture medium did not exceed 0.15% and did not affect the cells.

Before analyzing the egg yolk lipid profile, extracted fatty acids were converted into methyl derivatives using the method described by Morrison [10]. Lipid profile analysis was performed on a GCMS Shimadzu QP 5050 (Shimadzu, Kyoto, Japan), equipped with a SP-2560 capillary column (Sigma-Aldrich, St. Louis, MO, USA). The identification of fatty acid methyl esters was performed using a reference mixture of these compounds (FAME Mixture, Larodan Fine Chemicals, Stockholm, Sweden) and a mass spectral library (NIST 1.7).

### 2.2. Cell Cultures

The melanoma cells of line WM793 (CVCL_8787, ESTDAB) were cultured in the RPMI-1640 culture medium, with 10% fetal bovine serum (FBS) and antibiotic mixture. The BJ cells (ATCC) were cultured in the EMEM culture medium, with 10% FBS and antibiotic mixture. The cells were stored in an incubator (NuAire, Plymouth, MN, USA) at 37 °C in a 5% CO2 atmosphere. The culture medium was replaced with fresh medium every 2–3 days, passage was performed when 80% confluence was reached.

### 2.3. Cytotoxicity Analysis

The WM793 cells were seeded into 96-well plates at 9 × 10^3^ cells per well and cultured in 150 μL of culture medium for 24 h. The medium was then replaced with fresh medium containing mixture of fatty acids, isolated from chicken egg yolk enriched in CLA isomers by biofortification (FA-CLA) or mixture of fatty acids, isolated from chicken egg yolk unenriched in CLA (FA) and cultured for 24 h. The cytotoxicity assessment of the fatty acid mixture extracted from the unenriched and CLA-enriched chicken egg yolk was performed using the Cytotoxicity Detection Kit (LDH) (Roche, Basel, Switzerland) according to the protocol provided by the manufacturer.

### 2.4. Proliferation Analysis

The cells (WM793, BJ) were seeded into 96-well plates at 9 × 10^3^ cells per well and cultured in 150 μL of culture medium for 24 h. The culture medium was then replaced with the fresh medium containing FA-CLA or FA and cultured for another 24, 48 and 72 h. The control of the experiment was cells grown in the standard culture medium. The cell proliferation was determined using a commercially available Cell Proliferation ELISA kit, BrdU (Roche, Basel, Switzerland), according to the protocol provided by the manufacturer.

### 2.5. Apoptosis Assessment

To determine the effect of the tested fatty acid mixtures on apoptosis of the WM793, cells were seeded into 12-well plates at 8 × 10^4^ cells per well and cultured in 2 mL of culture medium for 24 h. The medium was then replaced with the fresh medium containing FA and FA-CLA and cultured for 48 h. As a positive control for apoptosis, the cells treated with 1.5 µM staurosporine for 2 h were used. The apoptosis analysis was performed with Annexin-V-FLUOS Staining Hit (Roche, Basel, Switzerland) according to the protocol provided by the manufacturer. The assessment of apoptotic activity was performed with a Zeiss Axio Observer.Z1 fluorescence microscope (Zeiss, Oberkochen, Germany). The number of apoptotic cells was determined by counting all the cells in the field of view of the microscope and the apoptotic cells stained green, in the same area. The location of cell counting in a given well was chosen randomly. The cell counting activity in each experiment was performed 5 times.

### 2.6. Gene Expression Analysis

In the experiment, the WM793 cells were seeded into 6-well plates at a rate of 2 × 10^5^ cells per well and cultured in 2 mL of culture medium for 24 h. The medium was then replaced with the fresh medium containing FA-CLA, FA, and cultured for another 48 h. The experimental control was the cells grown in the standard culture medium. After incubation, the total RNA was isolated from the cells with the use of Total RNA Mini Plus kit (A & A Biotechnology, Gdynia, Poland), according to the protocol provided by the manufacturer. The reverse transcription reaction was then performed using the RevertAid First Strand cDNA Synthesis Kit (Thermo Scientific, Waltham, MA, USA), according to the protocol received from the manufacturer. The material thus prepared was used to perform real-time PCR reactions using the commercially available TaqMan™ Array Human c-Myc and Apoptosis Kit (Thermo Scientific, Waltham, MA, USA). The analysis was conducted respectively on a StepOne System thermocycler (Thermo Scientific, Waltham, MA, USA) using StepOne Software v2.3 (Thermo Scientific, Waltham, MA, USA).

### 2.7. Protein Expression Analysis

The WM793 cancer cells were seeded into dishes with 1 × 10^6^ cells and incubated in 6 mL of a culture medium and cultured for 24 h. After the incubation, the medium was replaced with the fresh medium containing FA-CLA or FA sequentially and cultured for another 48 h. The experimental control was the cells grown in the standard culture medium and treated with 1.5 µM staurosporine for 2 h. After 48 h, the cells were lysed using Cell Lysis Buffer (Cell Signaling Technology, Danvers, MA, USA) and for protein fractions (cytoplasmic and membrane fractions) the Cell Fractionation Kit (Cell Signaling Technology, Danvers, MA, USA) was used according to the protocol. The determination of the amount of protein in the cell lysates was performed using the Pierce™ BCA Protein Assay Kit (Thermo Scientific, Waltham, MA, USA) and the Multiscan GO Microplate Reader (Thermo Scientific, Waltham, MA, USA). After determining the amount of protein in the lysate, the separation was performed with the use of electrophoresis. The separation was performed on an acrylamide gel (Sigma-Aldrich, ST. Louis, MO, USA). The separation was conducted in electrophoresis buffer (3.03 g Trizma Base, 14.4 g glycine, 1 g SDS, made up to 1 L with water), for 70 min, at 100 V. After the electrophoresis, the protein wet transfer was performed from the gel onto a nitrocellulose membrane (Bio-Rad, Hercules, CA, USA), in buffer (3.03 g Trizma Base, 14.4 g glycine 200 mL methanol, supplemented with water to vol. 1 L.). The transfer was conducted for 70 min using a voltage of 100 V and a current of 250–350 mA. The membranes after the transfer and blocking (5% milk) were incubated for one hour with primary antibodies (anti-caspase 12 #2202, anti-PARP #9542, anti-PARP cut #9548, anti-cytochrome C #11940, anti-BID #2002, anti-caspase 3 #14220, anti-caspase 3 cut #9664, anti-PUMA #12450, anti-GAPDH #5174, anti-β-actin #8457, anti-FADD #2782, anti-HtrA2/Omi #9745, Cell Signaling Technology, Danvers, MA, USA) at room temperature. After the incubation, the membranes were incubated for one hour with horseradish peroxidase-labelled goat anti-rabbit or anti-mouse secondary antibodies (#7074, #7076 Cell Signaling Technology, Danvers, MA, USA). Clarity™ Western ECL Substrate (Cell Signaling Technology, Danvers, MA, USA) was used for the signal detection. The membrane was transferred to a darkroom where visualisation of the detected protein was performed. The following were used for visualisation: photographic film, developer and fixer (Thermo Scientific, Waltham, MA, USA).

### 2.8. PathScan Protein Analysis

The protein lysates from whole cells were used for the experiment, as for Western blot analysis and the PathScan^®^ Stress and Apoptosis Signaling Antibody Array Kit (Cell Signaling Technology, Danvers, MA, USA). The experiment was performed according to the protocol provided by the manufacturer

### 2.9. Statistical Analysis

The results of the analyses were presented as mean results of the sums of biological and technical replicates. The statistical analysis of the results was performed with the statistical and analytical software package STATISTICA version 13 (StatSoft, Tucksa, Poland) and GraphPad Prism version 6.01 (GraphPad Software, San Diego, CA, USA).

## 3. Results

### 3.1. Profile Analysis of an Extracted Mixture of Fatty Acids

The analysis of the composition of the fatty acid mixture extracted from egg yolks obtained from Hy-Line Brown laying hens is shown in Table 1.

In the fatty acid samples enriched—by biofortification—with CLA isomers (FA-CLA), 12 different fatty acids were identified. Hexadecanoic acid (C16:0) accounted for the largest proportion (35.52%), followed by (Z)-9-octadecaenoic acid (C18:1n9) (26.11%) and octadecanoic acid (C18:0) (18.50%). In contrast, saturated fatty acids (SFA) constituted the largest group (54.98%). Additionally, in the samples, cis-9, trans-11 CLA and trans-10, cis-12 CLA isomers were identified, whose share in the total pool of fatty acids was 1.64 and 0.55%, respectively. In contrast, in the samples containing the FA mixture, 10 different fatty acids were identified, of which (Z)-9-octadecaenoic acid accounted for the largest proportion (42.51%), followed by hexadecanoic acid (C16:0) (26.63%) and (Z, Z)-9-octadecadienoic acid (C18:2n6) (17.23%). Monounsaturated fatty acids (MUFA) were the largest group (45.35%). No cis-9, trans-11 CLA and trans-10, cis-12 CLA isomers were identified in the samples.

The comparison of the composition of the FA-CLA and FA acid mixtures samples shows that significant statistical differences were found in the proportion of almost all the identified fatty acids, but these differences were non-unidirectional. The only fatty acid whose content was similar in both samples was (all-Z)-5,8,11,14-eicosatetraenoic acid. The total proportion of SFA and MUFA acids in the FA-CLA samples was significantly higher and lower, respectively, compared to the FA samples. Despite the presence of CLA isomers in the FA-CLA samples, which were not identified in the FA samples, the difference in the total amount of PUFAs of both samples was not statistically significant.

### 3.2. Selection of the Optimum, Non-Toxic Concentration Range of the Fatty Acid Mixture

**Figure 1:**
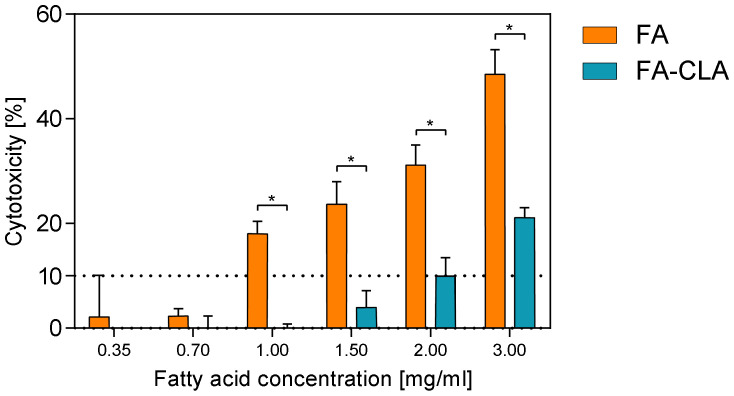
Effect of a mixture of fatty acids extracted from CLA-enriched and non-enriched yolks on cytotoxicity of human cancer cells line WM793. The Mann–Whitney U test was used for statistical analysis, *—statistically significant differences between FA-CLA and FA samples, at a significance level of *p* ≤ 0.05. The results are presented as mean, ± SD for *n* = 15.

The cytotoxic effect of a mixture of fatty acids, extracted from CLA-enriched and non-enriched chicken egg yolks, in concentrations ranging from 0.35 to 3.0 mg/mL of the culture medium, against human melanoma cell line WM793, after 24 h of the incubation, is shown in Figure 1.

Both fatty acid mixtures, the FA-CLA and FA, in concentrations of 0.35–0.70 mg/mL, had no toxic effect on the cancer cells of line WM793. Moreover, within this concentration range there are no statistically significant differences in the effect in question were observed between FA-CLA and FA mixtures. The increase of toxicity of FA-CLA mixture up to 10%, against WM793 cells, was observed at the concentration of 2 mg/mL, and at the highest analysed concentration (3 mg/mL) cytotoxicity was 21%. In contrast, an increase in the concentration of the fatty acid mixture, extracted from egg yolk not enriched in CLA, above 0.70 mg/mL, resulted in a sharp increase in the level of cytotoxicity from 2 to 18% for the concentration of 1 mg/mL, and to almost 50% at the FA mixture concentration of 3.0 mg/mL. The FA-CLA mixture, compared to the FA, had significantly less cytotoxicity against human melanoma cell line WM793 in the concentration range of 1.0–3.0 mg/mL.

On this basis, the concentrations in the range of 0.35–0.70 mg/mL were chosen for further studies of the effects of the fatty acid mixture extracted from egg yolks enriched and not enriched with CLA, on melanoma cancer cells of line WM793 and normal fibroblast line BJ.

### 3.3. Effects of a Fatty Acid Mixture on Proliferation of WM793 Cancer Cells and Normal BJ Cells

**Figure 2:**
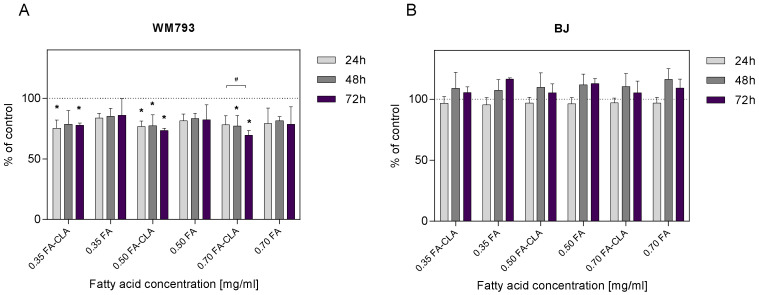
Effect of a mixture of fatty acids extracted from CLA-enriched and non-enriched yolks on the proliferation of human cancer cells of line WM793 (**A**) and normal line BJ (**B**). Statistical significance of differences was assessed using the Mann–Whitney U test at a significance level of *p* ≤ 0.05. The results are presented as mean, ± SD for *n* = 15. *—Differences statistically significant relative to the control sample, ^#^—differences statistically significant between time periods.

The WM793 cells treated with a mixture of CLA-enriched and non-CLA-enriched fatty acids showed differential proliferative potential (Figure 2A). The addition of a mixture of fatty acids extracted from CLA-enriched egg yolks, at a concentration of 0.35 mg/mL, to the cell cultures after 24 h of the incubation resulted in a statistically significant (*p* ≤ 0.05) reduction in cell proliferation of 24.65% compared to the control sample. This effect persisted at 48 and 72 h of the incubation, as the reduction in proliferation was 21.29 and 22.30%, respectively. The addition of FA-CLA mixture, at a concentration of 0.50 mg/mL, to the culture after 24 h resulted in a statistically significant (*p* ≤ 0.05) reduction in proliferation of 23.20% compared to control cells. The reduction in proliferation was maintained over the next 24 and 48 h at 22.63 and 26.42%. Similarly, the addition of FA-CLA mixture, at a concentration of 0.70 mg/mL, resulted in a 21.75% reduction in proliferation after 24 h of incubation, and further 48 and 72 h incubation of the cells resulted in a statistically significant (*p* ≤ 0.05) reduction in proliferation compared to the control sample by 22.77 and 30.54%, respectively.

The addition of fatty acid mixture extracted from egg yolk not enriched with CLA, at a concentration of 0.35 mg/mL, to the cell cultures resulted in a 16.22% reduction in proliferation, sustained over the next 24 and 48 h at 14.78 and 13.99%, respectively, compared to the control sample. Increasing the FA concentration to 0.50 mg/mL resulted in an 18.47% reduction in cell proliferation after 24 h of incubation, 16.58% after 48 h and 17.69% after 72 h. The addition of the FA mixture at a concentration of 0.7 mg/mL to WM793 cell cultures resulted in a decrease in proliferation after 24 h of incubation by 20.71%, after 48 h by 18.39%, and after 72 h by 21.38%. The observed reduction in cell proliferation under the influence of the FA mixture at the tested concentrations, at different time intervals, was not statistically significant (*p* > 0.05). Fatty acids extracted from non-enriched yolk showed only a tendency (*p* ≤ 0.1) to decrease the proliferation of human melanoma cell line WM793.

As shown in Figure 2B, the FA-CLA and FA mixtures at concentrations of 0.35, 0.50 and 0.70 mg/mL did not inhibit the proliferation of human normal fibroblast line BJ cells. Moreover, after 48 and 72 h of the incubation of cells with the FA-CLA mixture, at the three concentrations analysed, a non-significant increase in proliferation was observed from 5.19 to 10.35%, and for the FA mixture from 7.31 to 16.58%.

### 3.4. Evaluation of Cell Apoptosis Induced by the Test Mixture of Fatty Acids

**Figure 3:**
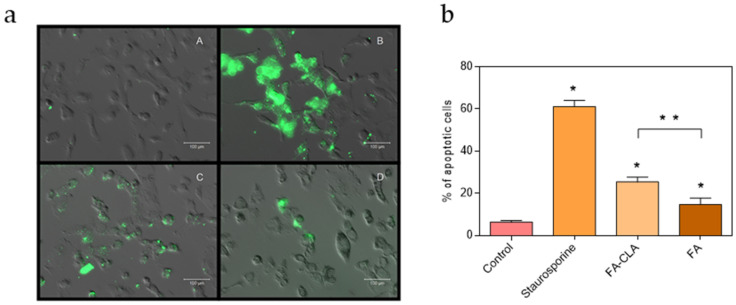
Detection of apoptotic cells with the Annexin-V-FLUOS Staining Kit Culture conditions of WM793 cells: A—control sample, B—staurosporine at a concentration of 1.5 µM, C—fatty acid mixture extracted from CLA-enriched chicken egg yolk, D—fatty acid mixture extracted from non-CLA-enriched chicken egg yolk. Apoptotic cells—green colour (**a**). Effect of a mixture of fatty acids extracted from egg yolk enriched and not enriched in CLA on apoptosis of human cancer cells line WM793. The graph indicates the percentage of apoptotic cells relative to all the cells cultured under each condition. The statistical significance of the differences, with respect to the control sample, was assessed by the Mann–Whitney U test with a significance level of *p* ≤ 0.05 (*) and *p* ≤ 0.01 (**) (**b**) The results are presented as mean, ± SD for *n* = 4.

The number of apoptotic cells varied according to the conditions under which the cells were cultured (Figure 3). In the control culture samples, apoptotic cells accounted for 6.4% of all cells. The addition of a mixture of fatty acids enriched in CLA to the cell culture, at a concentration of 0.50 mg/mL, resulted in a significant increase, compared to the control sample, in the number of apoptotic cells up to 25.5%. Moreover, the number of apoptotic cells in the culture containing the FA-CLA mixture was significantly higher than in the culture containing the FA mixture, already at a significance level of *p* ≤ 0.01. The incubation with the fatty acid mixture not enriched in CLA, at a concentration of 0.50 mg/mL, significantly (*p* ≤ 0.05), compared to the control sample, increased the number of apoptotic cells to 14.6%.

### 3.5. Gene Expression Analysis

The initiation of apoptosis is strictly regulated by activation mechanisms, and its initiation inevitably leads to cell death [11]. Most often it occurs through one of two pathways, the intrinsic (mitochondrial) or extrinsic (receptor) pathway. The intrinsic pathway is activated by intracellular signals generated when cells are stressed and depends on the release of proteins from the mitochondrial mesothelial space. The intrinsic pathway of apoptosis can be initiated in response to binding to nuclear receptors by glucocorticoids, heat, radiation, nutrient deficiency, viral infections, hypoxia, and increased intracellular calcium concentration [12]. The key proteins involve in this pathway are as follows: proteins from the Bcl-2 family (Bcl-2, Bad, Bax, Bid) and cytochrome C and APAF-1. The extrinsic pathway is activated by extracellular ligands that bind to cell surface death receptors, leading to the formation of a signaling complex that induces death. Death receptor mediated apoptosis begins with, among others, the Fas proteins and TNFR. Regardless of which signal activates apoptosis (internal or external), caspases are ultimately activated. Activation of caspases leads to DNA fragmentation, degradation of cytoskeletal and nuclear proteins, protein cross-linking, formation of apoptotic bodies, expression of ligands for phagocytic cell receptors and finally uptake by phagocytic cells [13].

The study showed that the fatty acid mixtures, both FA-CLA and FA, significantly increased or decreased the expression of the majority of the genes tested. At the same time, the expression of the same four genes was not detected in any of the experimental samples tested. These observations concerned the *CASP9*, *CDKN2A*, *FASLG*, and *IGF1* genes.

Compared to the control trial, the fatty acid mixture extracted from CLA-enriched egg yolks significantly (*p* ≤ 0.05) decreased the expression of only one gene—*YWHAH* (0.871-fold decrease), while it significantly increased the expression of the following seven genes: *BID* (3.123-fold increase)—encoding the Bid protein, which induces cytochrome C release from the mitochondrion to the cytosol; *CASP3* (1.132-fold increase)—encoding caspase 3; *FAS* (1.369-fold increase)—encoding the Fas receptor protein, involved in the receptor-mediated apoptosis pathway; *HRAS* (1.672-fold increase)—encoding the H-Ras protein, which regulates the cell cycle; *MYC* (2.174-fold increase)—encoding c-Myc protein, regulating cell proliferation, growth and apoptosis; *TP53* (1.256-fold increase)—encoding p53 protein, regulating DNA repair and apoptosis induction, and *BAX* gene (1.269-fold increase)—encoding Bax protein, acting as an activator of apoptosis (Table 2).

In contrast, the fatty acid mixture, extracted from egg yolks not enriched in CLA, significantly (*p* ≤ 0.05) decreased the expression of four genes: *BAX* (0.882-fold decrease) (in contrast to the fatty acid mixture extracted from CLA-enriched egg yolk); *NRAS* (0.891-fold decrease)—encoding the N-Ras protein, which regulates the cell cycle; *YWHAH* (0.739-fold decrease) and *YWHAZ* (0.893-fold decrease) genes—encoding 14-3-3 family proteins, which regulate many processes, including the cell cycle and apoptosis. At the same time, the FA mixture did not significantly increase the expression of any of the analysed genes.

Comparing the changes in gene expression in the cells cultured in the environment of the tested fatty acid mixtures, it was found that the FA-CLA mixture increased the expression of *BAX*, *CASP3*, *FAS*, *HRAS*, *MYC*, *TP53*, *YWHAZ* genes more strongly (*p* ≤ 0.05) than FA. In contrast, the FA-acid mixture inhibited the expression of the *YWHAH* gene more effectively (*p* ≤ 0.05) than the FA-CLA mixture.

### 3.6. Protein Expression Analysis

**Figure 4:**
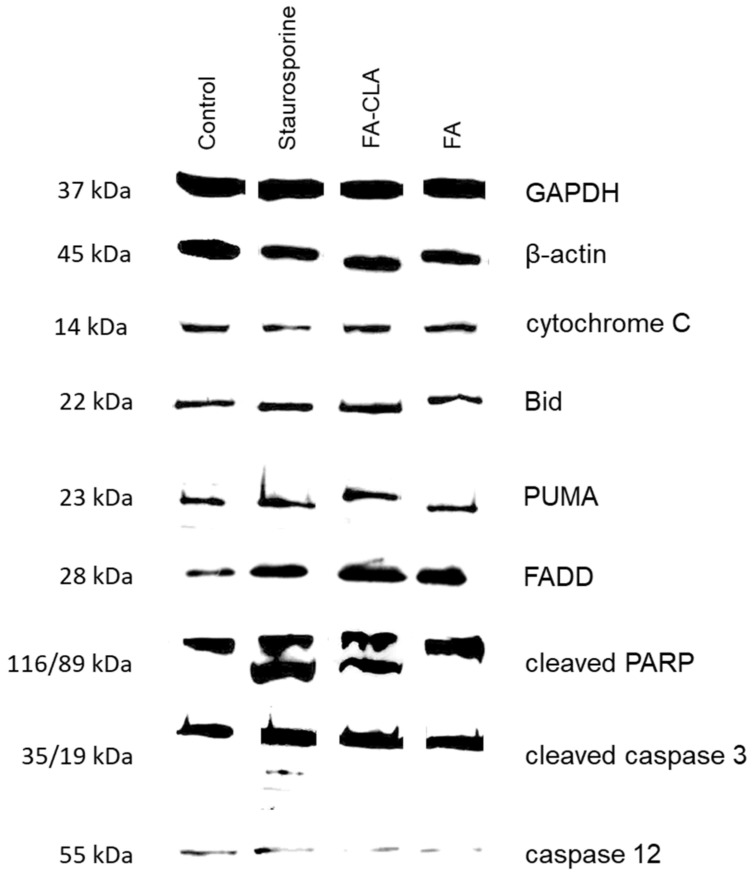
Expression of apoptotic proteins in cancer cells of the WM793 line when exposed to a mixture of fatty acids extracted from yolk enriched and not enriched in CLA.

As a first step, the expression of seven apoptotic proteins from the whole cell lysates (caspase 12, PARP, cytochrome C, Bid, caspase 3, PUMA, FADD) was analysed (Figure 4).

**Figure 5:**
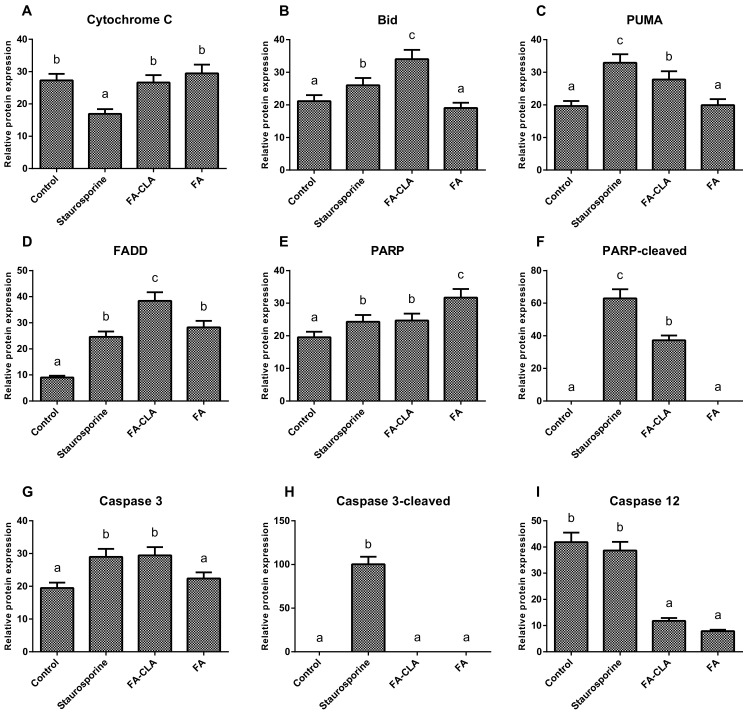
Effect of a mixture of fatty acids extracted from CLA-enriched and non-enriched yolk on the expression of individual apoptosis-related proteins in cancer cells of line WM793. The Tukey test was used for statistical analysis. Values marked with different letters are significantly different at *p* ≤ 0.05. (**A**–**I**) analyzed proteins. The results are presented as mean, ± SD for *n* = 4 in relation to untreated cells (control), a–c—means with the same letter are not significantly different.

The differences in the expression of cytochrome C (Figure 5A), both with respect to the cells of the control trial and between the trials treated with the FA-CLA and FA mixture, were not significant. The expression of the proapoptotic protein Bid (Figure 5B) increased significantly (*p* ≤ 0.05), as compared to the control trial, when a mixture of fatty acids extracted from egg yolks enriched in CLA was added to the cell cultures. Furthermore, the increase in Bid protein expression in the FA-CLA mixture medium was significantly greater (*p* ≤ 0.05) than when staurosporine was added to the cells. The mixture of fatty acids extracted from unenriched egg yolk (FA) did not alter Bid protein expression. A similar effect was achieved by analysing the expression of the PUMA protein (Figure 5C). The FA-CLA fatty acid mixture, similarly to staurosporine, significantly (*p* ≤ 0.05) increased its expression, while the FA mixture did not show an effect compared to the control. The fatty acids analysed significantly (*p* ≤ 0.05) increased the expression of FADD protein (Figure 5D) compared to the control trial. The expression level of this protein following the treatment with the FA-CLA mixture was three times higher compared to the control culture. The expression of FADD in the cells cultured in FA mixture and staurosporine medium was similar. PARP protein expression (Figure 5E) increased significantly (*p* ≤ 0.05) in all analysed cases, with respect to the control cell culture. Moreover, the protein expression level under the effect of the fatty acid mixture, extracted from egg yolks not enriched in CLA, was significantly (*p* ≤ 0.05) higher than under the influence of the FA-CLA mixture. In contrast, truncation (inactivation) of PARP protein (Figure 5F) occurred only under the influence of a mixture of fatty acids extracted from CLA-enriched egg yolk and staurosporine. The expression of caspase 3 (Figure 5G) increased significantly (*p* ≤ 0.05) in the cells cultured in the medium of a mixture of fatty acids extracted from CLA-enriched egg yolk and staurosporine, and the increase was comparable in both cases. Whereas, the expression level of caspase 3 in the cells under the influence of the FA mixture was comparable to the control trial. As shown in Figure 5H, caspase 3 activation (truncation) occurred only when staurosporine was added to the cells. The analysis of caspase 12 expression (Figure 5I) showed a significant (*p* ≤ 0.05) reduction in protein levels, compared to the control trial, in the cells cultured in medium supplemented with a mixture of fatty acids extracted from chicken egg yolk, both enriched and unenriched in CLA.

**Figure 6:**
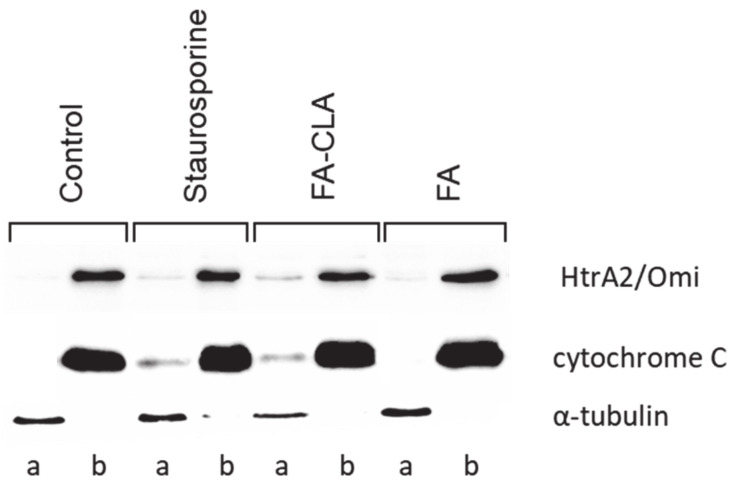
Expression of apoptotic proteins in different cell fractions, in the WM793 cancer cells, under the influence of a mixture of fatty acids extracted from yolk enriched and not enriched in CLA. a—cytosolic fraction, b—membrane fraction.

The second stage of the experiment consisted in examining the changes in cytochrome C and HtrA2/Omi content in the cell fractions (membrane and cytosolic) under the influence of the tested fatty acid mixtures (Figure 6).

**Figure 7:**
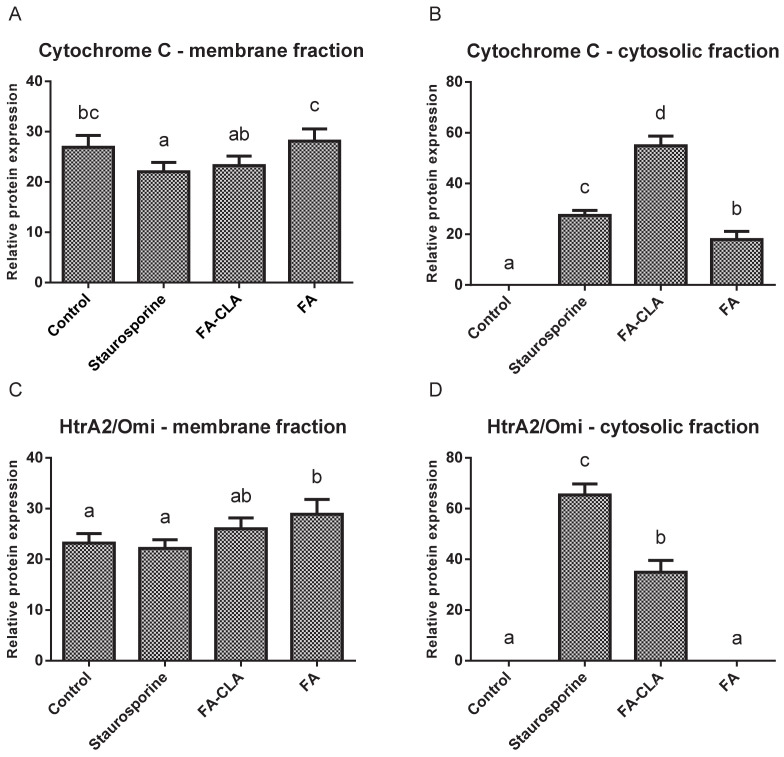
Effect of FA-CLA and FA mixture on the expression of individual proteins in cell lysates of membrane and cytosolic fractions obtained from cancer cells of line WM793. The Tukey test was used for statistical analysis values marked with different letters are significantly different at *p* ≤ 0.05. (**A**–**D**) analyzed proteins. The results are presented as mean, ± SD for *n* = 4 in relation to untreated cells (control), a–d—means with the same letter are not significantly different.

Cytochrome C levels (Figure 7A) in the mitochondrial fraction were comparable in all trials. A small but statistically significant (*p* ≤ 0.05) reduction in the amount of protein, compared to the control trial, was observed in the cells treated with staurosporine.

Meanwhile, no cytochrome C was detected in the cytoplasmic fraction of the cells cultured in standard medium (Figure 7B). This protein was detected in the remaining experimental samples and its amount, relative to the control trial, differed significantly (*p* ≤ 0.05) in each case. The highest amount of cytochrome C was observed in the lysate obtained from the cells cultured in medium with a mixture of fatty acids extracted from CLA-enriched chicken egg yolk, and the least in those cultured with FA-CLA mixture. The differences found were statistically significant.

Similar to cytochrome C, HtrA2/Omi protein in the mitochondrial fraction was detected at similar levels in all samples analysed (Figure 7C). Only in the cells cultured in medium with a fatty acid mixture extracted from egg yolk not enriched in CLA, the level of HtrA2/Omi was significantly (*p* ≤ 0.05) higher compared to the control trial.

In contrast, the analysed protein was not detected in the cytoplasmic fraction of the control cells and those cultured in medium with FA mixture. This protein was identified in lysates from the cells treated with the FA-CLA mixture (Figure 7D).

### 3.7. PathScan Protein Activation Analysis

**Figure 8:**
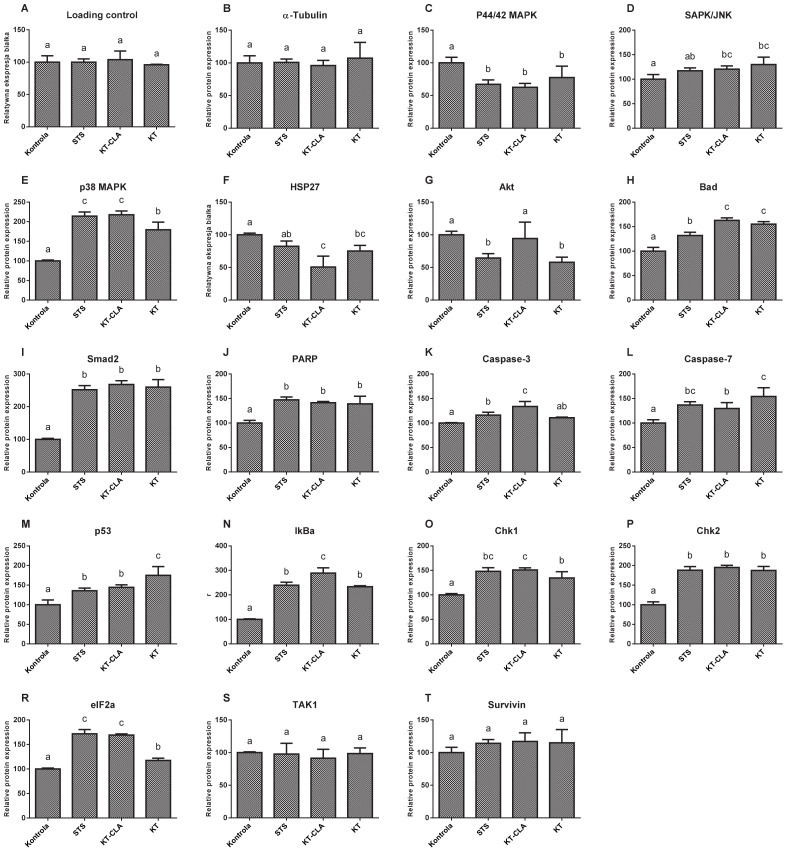
Effect of a mixture of fatty acids extracted from CLA-enriched and non-enriched yolk on the expression of individual apoptosis- and stress-related proteins in cancer cells of the WM793 line. The Tukey test was used for statistical analysis. Values marked with different letters are significantly different at *p* ≤ 0.05. (**A**) loading control, (**B**) reference protein, (**C**–**T**) analyzed proteins. The results are presented as mean, ± SD for *n* = 4 in relation to untreated cells (control), a–c—means with the same letter are not significantly different.

The levels of MAP protein kinases, ERK1 and ERK2 (Figure 8C), were lower by 37.47% in the cells cultured in CLA-enriched fatty acid mixture medium, and with respect to those cultured in non-CLA-enriched medium by 22.55%, compared to the control trial (*p* ≤ 0.05). In contrast, the expression of SAPK/JNK kinase (Figure 8D), in the cells cultured in the medium of both, FA-CLA and FA, was significantly (*p* ≤ 0.05) higher, by 20.43% and 29.92%, respectively, compared to the control culture cells. The level of MAPK kinase p38 (Figure 8E), in all samples tested was significantly higher with respect to the control conditions. Moreover, it increased by 117.9% after the addition of the FA-CLA mixture. The activation of HSP27 protein (Figure 8F) was highest in the lysate obtained from the cells of the control sample, as it decreased by 49.28% (*p* ≤ 0.05) and 24.88% (*p* ≤ 0.05), respectively, after the addition of FA-CLA and FA mixture to the culture medium. The activation of the Akt protein (Figure 8G), after the addition of FA-CLA decreased by 5.86%, but this decrease was not statistically significant (*p* > 0.05). A significant decrease in the Aka activation (42.2%) occurred after the addition of FA. The FA-CLA and FA fatty acid mixtures analysed increased significantly (*p* ≤ 0.05) the activation of Bad protein (Figure 8H) by 62.71 and 55.09%, respectively, and Smad2 protein (Figure 8I), y by more than 150% (*p* ≤ 0.05) in all analysed samples. Activation (truncation) of PARP protein in cancer cells of line WM793 under FA-CLA and FA mixture treatment, compared to the control conditions, increased significantly (*p* ≤ 0.05) by 39 and 21%, respectively (Figure 8J). The activation of executive caspases 3 and 7 (Figure 8K,L) in the melanoma cells of line WM793 significantly (*p* ≤ 0.05) increased under the influence of FA-CLA mixture by 33.72% and 16.09%, respectively. In contrast, the FA mixture enhanced the activation of caspase 7 alone by 54.26% (*p* ≤ 0.05) compared to the control culture. The activation levels of p53 (Figure 8M), Iκβα (Figure 8N) Chk1 (Figure 8O) and Chk2 (Figure 8P) proteins significantly (*p* ≤ 0.05) increased in all experimental samples. A similar direction of change was found for the eIF2a protein (Figure 8R). The FA-CLA mixture increased significantly (*p* ≤ 0.05) the expression of this protein by 96% and the FA mixture by 17.42%. The activation levels of TAK1 (Figure 8S) and survivin (Figure 8T), did not change significantly (*p* > 0.05) in all experimental samples.

## 4. Discussion

The treatment of cancer is difficult, expensive and not always effective. It usually consists of invasive surgery and chemotherapy, which is not neutral to the body. The best prevention against cancer is to conduct research leading to its quick and early detection, which significantly increases the chances of being cured, as well as a “healthy lifestyle”. Cancer risk can be modified by, among other things, maintaining a healthy body weight, eating adequate amounts of low-sugar vegetables and fruits, and avoiding xenobiotics and certain stimulants. An important key element of cancer prevention is the enrichment of the diet with bioactive compounds that have an inhibitory effect on growth, proliferation and metastasis, as well as assisting the immune system in eliminating altered cells at an early stage of cancer development. Early prevention of cancer can furthermore reduce health care and treatment costs [14].

The aim of this article is to demonstrate the possible health-promoting properties of CLA-enriched eggs as a functional food that can be used in cancer prevention. Hen eggs biofortification is a simple and natural process that takes place through the diet. Our previous studies [8] examined the effect of individual CLA isomers: cis-9, trans-11 and trans-10, cis-12 on the MCF-7 cells. It has been shown that synthetic CLA isomers are less potent than the fatty acid mixture extracted from CLA-enriched egg yolks in inhibiting proliferation of cancer cells. It can be concluded that composition of chicken eggs and changing the proportion of SFA and MUFA may enhance the anti-cancer properties of CLA and induce apoptosis.

In an experiment carried out on the melanoma cancer cells of the WM793 line, a significant (*p* ≤ 0.05) reduction in proliferation, ranging from 21.29 to 30.54%, was observed under the effect of a mixture of fatty acids containing CLA isomers, at concentrations of 0.35, 0.50 and 0.70 mg/mL of the culture medium with no effect on normal cells. Progressive inhibition of proliferation within 72 h was observed with the above mixture, at concentrations of 0.50 and 0.70 mg/mL. The mixture of fatty acids from egg yolk unenriched with CLA, in the tested concentration ranges, did not affect significantly the proliferation of cancer cells. Similar results were obtained in the study of Koronowicz et al. [15], in which the proliferation of MCF-7, breast cancer, decreased by 40%. According to Ochoa et al. [16], the treatment of PC3 prostate cancer cells with synthetic isomers of CLA (trans-10,cis-12 CLA) reduces their proliferation by up to 30%.

The observed effect of the tested fatty acid mixtures on decreasing the proliferation of cancer cells of line WM793 may be due to at least two reasons.

The method of cell proliferation analysis using bromodeoxyuridine (BrdU) indirectly illustrates the biosynthesis of new DNA. Thus, the downregulation of the signal, derived from BrdU, towards the control cells may be due to a reduction in DNA synthesis, thus inhibiting proliferation, or to cell death, not necessarily due to inhibition of proliferation. The cell death could be caused by apoptosis or necrosis, which has been ruled out in earlier experiments. The possibility of inhibition of proliferation with concomitant apoptosis of the cancer cells is also plausible, and in view of anticancer prevention, this would be a most desirable result.

The imaging of the cells with annexin V and propidium iodide, using a fluorescence microscope, is a simple and rapid method to differentiate between two cell death processes: apoptosis and necrosis. The analysis of the number of live and apoptotic cancer cells of the WM793 line, untreated—negative control, treated with staurosporine (1.5 µM)—positive control and with a mixture of fatty acids extracted from unenriched and CLA-enriched yolk, at a concentration of 0.50 mg/mL, showed the ability of the latter to induce apoptosis. The mixture of fatty acids extracted from chicken egg yolk not enriched in CLA induced apoptosis of about 15% of the cells, while the mixture of fatty acids extracted from chicken egg yolk enriched in CLA induced apoptosis of 25% of the cells.

The results obtained in this experiment are consistent with the results of the proliferation analysis, which may suggest that the reduction observed was related more to cell death than to a reduction in the number of divisions.

Miller et al. [17] showed that fatty acids extracted from milk enriched in the cis-9, trans-11 isomer of CLA induce cell apoptosis colon cancer cells of the SW480 line, characterized by an increase in annexin V signal, which was not observed with fatty acids extracted from unenriched milk.

The main marker of apoptosis in cells is the activation of caspases. A particular caspase whose activation irreversibly leads to cell death is caspase 3. The activation of caspase 3 takes place in both mitochondrial and receptor-mediated pathways of apoptosis. In the former case, the activation occurs by release of cytochrome C from the mitochondrial membrane into the cytoplasm and formation of the apoptosome, in the latter by the activation of death receptors and proteins FADD and TADD and consequently caspase 8, for which procaspase 3 is a substrate [18].

The melanoma cells of the WM793 line treated with FA-CLA showed a significant increase in caspase 3 levels compared to the control culture cells (Figure 5G). This result confirms the observations made during gene expression analysis (Table 2). Moreover, the FA-CLA mixture, in addition to increasing caspase 3 levels in the cells, led to caspase 3 activation (truncation) (Figure 8K). There was also an increase in caspase 7 activity (Figure 8L), and an increase in the expression of caspase 12 (Figure 8L), activated by the damage to the endoplasmic reticulum.

As demonstrated, the expression of the *CYCS* gene (Table 2), encoding cytochrome C in the WM793 cells, did not change under the influence of the tested fatty acid mixtures. An analogous result was obtained by analysing protein expression by Western blot. More importantly, only the analysis of cytochrome C levels in individual protein fractions, i.e., membrane and cytosolic, showed that the fatty acid mixture enriched in the CLA causes the movement of this protein from the mitochondrial membrane to the cytoplasm (Figure 7A,B), which is a key element of apoptosis. The release of cytochrome C from the mitochondrion into the cytoplasm under the influence of a mixture of FA-CLA fatty acids and synthetic isomers in their work was described by Koronowicz et.al. [19] and Miglietta et al. [20], respectively.

In the study, the CLA-enriched fatty acid mixture induced the release into the cytoplasm from mitochondrial membranes of a second protein, HtrA2/Omi (Figure 7C,D), which, similarly to cytochrome C, is an important signal for enhancing apoptosis. The expression of the Smad2 protein, which controls the release of cytochrome C and the translocation of Bax and Bad proteins to mitochondrial membranes, was also increased. The expression of *BAX* gene significantly increased (Table 2).

One of the substrates for caspase 3 is the PARP protein. The fatty acid mixtures tested in the analysis significantly (*p* ≤ 0.05) increased PARP protein expression (Figure 5E). The increase in the PARP protein level may suggest the anti-apoptotic properties of the mixtures, as this protein is responsible for repairing damaged DNA, which may lead to the survival of the damaged cells. However, as the protein content increased, the degree of PARP truncation also increased (Figure 5F), and fragments of truncated PARP protein are potent activators of the mitochondrial apoptosis pathway [21].

A protein involved in death receptor-dependent apoptosis is FADD. The expression of the gene (Table 2) encoding this protein in cancer cells of line WM793, under the influence of a mixture of fatty acids, both enriched and not enriched in CLA, did not change significantly, but there was a tendency (*p* ≤ 0.1) to increase it under the influence of the FA-CLA mixture, which was confirmed by Western blot (Figure 5D).

The link between receptor-mediated and mitochondrial apoptosis is the Bid protein [22]. As shown above, the expression of the *BID* gene in cancer cells of the WM793 line, treated with a fatty acid mixture extracted from CLA-enriched chicken egg yolks, increased 3-fold (Table 2). The increase in gene expression correlated with an increase in Bid protein levels in the cells (Figure 5B).

The FA-CLA mixture was shown to increase the expression of the *TP53* gene (Table 2) and the levels of proteins associated with p53-dependent apoptosis in the cancer cells of the WM793 line. The fatty acid mixture containing CLA increased protein levels of p53, PUMA, p38, SAPK/JNK, Chk1 and Chk2.

The fatty acid mixture, isolated from chicken egg yolk, both CLA-enriched and non-enriched, did not affect the levels of the anti-apoptotic protein TAK1, survivin, and decreased the protein levels of HSP27. Survivin and HSP27 are proteins that block the release of cytochrome C from the mitochondrial membrane, which consequently leads to the survival of cells [23,24].

In the authors work, it was shown that the analysed fatty acid mixtures, both enriched and non-enriched in CLA, cause a decrease in the levels of Akt and p44/42 MAPK proteins in the WM793 cancer cells, responsible for cell growth, proliferation and differentiation. The Akt protein is also responsible for phosphorylation of the Bad protein, which in this form, bound to 14-3-3 proteins, remains inactive in the cytosol [25].

In turn, the effect of CLA isomers contained in fatty acid mixtures enriched by biofortification on the expression of genes related to cell proliferation is not obvious. It seems, therefore, justified to conduct further studies on their influence on the growth and proliferation of cancer cells, with particular emphasis on the role of the *MYC* gene, encoding the c-MYC protein.

## 5. Conclusions

**Figure 9:**
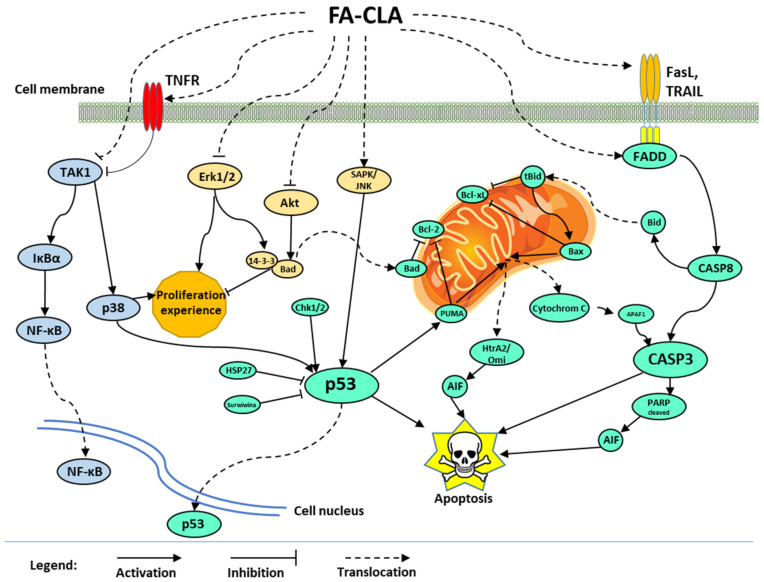
Proposed mechanism of apoptosis of melanoma cells of line WM793 under treatment with a mixture of fatty acids extracted from egg yolk enriched in CLA.

The mechanism of apoptosis of the human cancer cell line WM793 proposed in this study, influenced by the recorded action of a mixture of fatty acids extracted from CLA-enriched chicken egg yolk, involves three signalling pathways (Figure 9).

In the first one, caspase 3 is activated by the release of cytochrome C from mitochondrial membranes into the cytoplasm. The release of this protein was caused by the inhibition of antiapoptotic proteins of the Bcl-2 family and activation of proapoptotic proteins such as Bad, Bax and PUMA. The activation of mitochondrial apoptotic pathway may also occur through p53 protein, whose expression significantly increased under the influence of FA-CLA mixture, or through the activation of death receptors and apoptosis signal transduction associated with the activation of Bid protein. During apoptosis, in addition to the release of cytochrome C, there was a release of HtrA2/Omi protein and the truncation of PARP protein, fragments of which are potent apoptotic factors. The increase in p53 protein activity was caused, among other things, by a decrease in the expression of survival proteins such as HSP27 and survivin.

The second signalling pathway whose activity was altered by the FA-CLA mixture was the Akt protein and Erk 1/2 kinase pathway. The inhibition of the activity of these proteins resulted in inhibition of cell proliferation and survival.

The third pathway affected by the FA-CLA mixture was the activation of NF-κB factor. NF-κB activation was associated with the TNF receptor-dependent protein TAK1 and IκBα. The effect of FA-CLA mixture on NF-κB factor activation requires further study.

The gene expression analysis (Table 2) showed a 2-fold increase in the expression of the *MYC* gene, which corkscrews the c-Myc protein, in cells of the WM793 line treated with the FA-CLA mixture, with respect to the control conditions. In turn, the role of c-Myc protein in the regulation of the cell proliferation and apoptosis treated with the CLA mixture is not clear. On the one hand, the c-Myc protein is responsible for decreasing cell proliferation and apoptosis, on the other hand, it can increase the proliferation and have an anti-apoptotic effect [26,27]. Further studies are needed to better understand the signalling pathways that depend on c-Myc protein activity under the influence of FA-CLA mixture.

## Figures and Tables

**Table 1:** Fatty acid profile of extracted chicken egg yolks enriched (FA-CLA) and non-enriched in CLA (FA).

Fatty Acid	FA-CLA (Group II)			FA (Group I)		
C14:0	0.75 ^b^	±	35.8	0.32 ^a^	±	0.08
C16:0	35.52 ^b^	±	1.37	26.63 ^a^	±	0.95
C16:1n9	0.20 ^b^	±	0.06	0.54 ^a^	±	0.23
C16:1n7	0.85 ^b^	±	0.27	2.29 ^a^	±	0.57
C17:0	0.21 ^b^	±	0.03	0.11 ^a^	±	0.03
C18:0	18.50 ^b^	±	0.56	8.74 ^a^	±	2.46
C18:1n9	26.11 ^b^	±	1.24	42.51 ^a^	±	2.69
C18:2n6	14.39 ^b^	±	0.88	17.23 ^a^	±	0.72
C18:3n6	0.64 ^b^	±	0.10	0.47 ^a^	±	0.15
C18:2 cis-9, trans-11 CLA	1.64	±	0.36	Nd		
C18:2 trans-10, cis-12 CLA	0.55	±	0.23	Nd		
C20:4n6	0.63 ^a^	±	0.49	1.18 ^a^	±	0.72
SFA	54.98 ^b^	±	0.60	35.8 ^a^	±	1.13
MUFA	27.16 ^b^	±	0.63	45.35 ^a^	±	1.33
PUFA	17.85 ^a^	±	0.30	18.88 ^a^	±	0.33

The results are presented as the proportion (%) of each fatty acid among all identified. The Tukey test was used for statistical analysis. The values marked in rows with different letters are significantly different at *p* ≤ 0.05. The results are presented as mean, ± SD for *n* = 6, a,b—means with the same letter are not significantly different, Nd—not detected.

**Table 2:** Expression of genes related to apoptosis and cell cycle in cancer cells of WM793 line exposed to a mixture of CLA-enriched and non-enriched fatty acids.

Gene	FA-CLA	FA
*AKT1*	1.038 ± 0.022	0.991 ± 0.078
*APAF1*	0.723 ± 0.055	0.814 ± 0.064
*BAD*	1.044 ± 0.079	1.277 ± 0.101
*BAX*	1.269 *^,b^ ± 0.096	0.882 *^,a^ ± 0.069
*BCL2*	1.123 ± 0.085	0.752 ± 0.059
*BID*	3.123 * ± 0.236	1.642 ± 0.129
*CASP3*	1.132 *^,a^ ± 0.086	1.066 ^b^ ± 0.084
*CASP8*	1.547 ± 0.117	1.244 ± 0.098
*CASP9*	Nd	Nd
*CDKN2A*	Nd	Nd
*CYCS*	0.903 ± 0.068	1.181 ± 0.093
*FADD*	1.547 ± 0.117	0.639 ± 0.050
*FAS*	1.369 *^,a^ ± 0.104	1.131 ^b^ ± 0.089
*FASLG*	Nd	Nd
*HRAS*	1.672 *^,a^ ± 0.127	1.192 ^b^ ± 0.094
*IGF1*	Nd	Nd
*IGF1R*	1.025 ± 0.078	0.967 ± 0.076
*KRAS*	0.522 ± 0.039	0.910 ± 0.072
*MYC*	2.174 *^,a^ ± 0.164	1.603 ^b^ ± 0.126
*NRAS*	0.945 ^b^ ± 0.071	0.891 *^,a^ ± 0.070
*RRAS*	1.118 ± 0.085	1.372 ± 0.108
*TP53*	1.256 *^,a^ ± 0.095	1.137 ^b^ ± 0.090
*YWHAB*	1.097 ± 0.083	1.106 ± 0.087
*YWHAE*	1.052 ± 0.080	1.111 ± 0.088
*YWHAG*	0.887 ± 0.067	1.042 ± 0.082
*YWHAH*	0.821 * ± 0.062	0.739 * ± 0.058
*YWHAQ*	1.166 ± 0.088	1.228 ± 0.097
*YWHAZ*	0.871 ^b^ ± 0.013	0.893 *^,a^ ± 0.070

Statistical significance of differences was assessed with the t-Student test, at the significance level of *p* ≤ 0.05. *—statistically significant differences with respect to the control trial, a,b—means with the same letter are not significantly different between FA-CLA and FA samples. The results are presented as mean, ±SD for *n* = 6, Nd—expression was not detected.

## Data Availability

Not applicable.
